# Unraveling Charge Transfer Mechanisms in Graphene–Quantum Dot Hybrids for High-Sensitivity Biosensing

**DOI:** 10.3390/bios15050269

**Published:** 2025-04-24

**Authors:** Shinto Mundackal Francis, Hugo Sanabria, Ramakrishna Podila

**Affiliations:** Department of Physics and Astronomy, Clemson University, Clemson, SC 29634, USA; shintof@clemson.edu (S.M.F.); hsanabr@clemson.edu (H.S.)

**Keywords:** graphene, quantum dots, biosensing, photoluminescence, field-effect transistor

## Abstract

Colloidal quantum dots (QDs) and graphene hybrids have emerged as promising platforms for optoelectronic and biosensing applications due to their unique photophysical and electronic properties. This study investigates the fundamental mechanism underlying the photoluminescence (PL) quenching and recovery in graphene–QD hybrid systems using single-layer graphene field-effect transistors (SLG-FETs) and time-resolved photoluminescence (TRPL) spectroscopy. We demonstrate that PL quenching and its recovery are primarily driven by charge transfer, as evidenced by an unchanged fluorescence lifetime upon quenching. Density functional theory calculations reveal a significant charge redistribution at the graphene–QD interface, corroborating experimental observations. We also provide a simple analytical quantum mechanical model to differentiate charge transfer-induced PL quenching from resonance energy transfer. Furthermore, we leverage the charge transfer mechanism for ultrasensitive biosensing to detect biomarkers such as immunoglobulin G (IgG) at femtomolar concentrations. The sensor’s electrical response, characterized by systematic shifts in the Dirac point of SLG-FETs, confirms the role of analyte-induced charge modulation in PL recovery. Our findings provide a fundamental framework for designing next-generation graphene-based biosensors with exceptional sensitivity and specificity.

## 1. Introduction

Colloidal quantum dots (QDs) have received significant attention in optoelectronic and sensing applications due to their high quantum yield, size-tunable band gap, narrow spectral emission, and robust photostability [1,2,3]. Their integration with two-dimensional (2D) materials, such as graphene, has opened new avenues for exploring light–matter interactions at the nanoscale [4,5]. Single-layer graphene (SLG) exhibits a linear energy dispersion at the *K* and *K’* points in the Brillouin zone with the Fermi energy (*E_F_*) at the Dirac point [6,7]. The ability to tune *E_F_* in SLG with simple electrostatic gating or through SLG-QD hybrids paved the way for SLG-field effect transistors (SLG-FETs), which have been widely used as sensors [8,9,10,11,12]. Given the broad applicability of hybrid SLG-QD architectures for biomedical sensing to next-generation photovoltaics, understanding their interaction mechanisms is of particular interest.

Graphene is known to perturb the photoluminescence (PL) emission from a fluorophore or QDs in its close vicinity [13,14,15]. Kasry et al. showed that graphene is an efficient quencher of PL emission, with a quenching efficiency higher than that of gold [16]. Brus et al. reported a high quench factor of ∼25 for an CdSe/ZnS QDs on SLG surface [17]. Similarly, Federspiel et al. demonstrated that the quenching of CdSe QDs on MgO/graphene substrates shows *d*^−4^ distance dependence [13].

The recovery of PL emission from graphene–QD hybrids has been used widely in biosensors [18,19,20,21,22,23]. The first-ever application of quenching followed by Förster resonance energy transfer (FRET) at the QD–graphene oxide (GO) interface for biomolecular detection was proposed by Dong et al. [24]. The sensing mechanism in such sensors relies on the disruption of quenching in the presence of the target analyte. In this methodology, it is proposed that when the analyte binds functionalized QDs immobilized on GO or SLG surface, the distance between the QDs and GO/SLG increases, significantly hindering FRET. Consequently, PL emission from QDs is recovered [25,26,27,28,29]. However, this distance-dependent PL modulation has remained a subject of debate, particularly given the inconsistencies in the type of graphene used (e.g., SLG or few-layer graphene, exfoliated graphene, graphene oxide or GO, reduced graphene oxide or rGO etc). Alternative studies have proposed that PL quenching in graphene–QD systems may be attributed to mechanisms such as charge transfer rather than energy transfer [30,31,32,33].

In this study, we aim to clarify the underlying mechanism of PL quenching and recovery in graphene–QD hybrid sensors. Utilizing SLG-FET measurements and time-resolved photoluminescence (TRPL) spectroscopy, we show that quenching occurs through rapid charge transfer before optical excitation. Our findings indicate that the fluorescence lifetime remains unchanged upon quenching, supporting the charge transfer-induced static quenching mechanism. To further elucidate this mechanism, we present a heuristic quantum mechanical model demonstrating how analyte interactions with either QDs, SLG, or both can alter charge transfer characteristics, leading to PL recovery. This model offers a simple understanding of the PL modulation in graphene–QD hybrids, which is crucial for the rational design of advanced sensing platforms.

Building on the charge transfer quenching mechanism, we utilized two well-characterized biological interactions—biotin–streptavidin and immunoglobulin G (IgG)-anti-IgG antibody recognition—to develop femtomolar biosensors with dual detection capabilities (optical and electrical). The biotin–streptavidin system is a model bioaffinity pair that is widely used in biosensors due to its exceptionally strong binding affinity (dissociation constant K_d_ ≈ 10^−15^ M). We show that when biotin interacts with streptavidin-functionalized CdSe QDs, the resulting biomolecular interactions alter the charge transfer dynamics between the QD and graphene, disrupting the quenching effect and restoring QD fluorescence. We observed a linear PL recovery with increasing biotin concentration, with significant signal enhancement at femtomolar concentrations, highlighting the ultrasensitive nature of the detection mechanism. Similarly, we investigated the detection of IgG, a key biomarker in immune response and disease diagnostics. The interaction between IgG and its corresponding anti-antibody (anti-IgG) conjugated with CdSe QDs leads to charge redistribution at the graphene interface, modulating both optical and electrical properties. Notably, FET measurements revealed a systematic shift in the Dirac point voltage (*V_CNP_*) with increasing IgG concentration, confirming the doping effect of biomolecules on the graphene channel. The recovery of PL intensity was highly sensitive to IgG concentrations as low as 0.5 fM, underscoring the ability of our sensor to detect minute molecular changes. We found little to no change in the decay time of anti-IgG conjugated QD/SLG hybrids in the presence of different IgG concentrations, further confirming the charge transfer mechanism.

## 2. Materials and Methods

### 2.1. Materials and Reagents

The SLG-FET sensor array (0.8 mm^2^), monolayer graphene on a 90 nm Si/SiO_2_ (10 mm^2^) and quartz substrate, all grown with chemical vapor deposition, were purchased from Graphenea Semiconductor SLU, San Sebastián, Spain. The SLG-GFET array (model: mGFET-4D) comprises 28 graphene channels with Au metal pads passivated with 50 nm Al_2_O_3_ via atomic layer deposition. The non-encapsulated electrode at the center of the chip enables liquid gating. CdSe QDs (Invitrogen, CA, USA, Cat. # Q10123MP), anti-human IgG antibody (Ab) (Abcam, MA, USA, Cat.# ab109489), biotin, bovine serum albumin (BSA), phosphate-buffered saline (PBS), and Tween20 were purchased from Thermo Fisher Scientific, San Jose, CA, USA. Sample preparation and CdSe QD- Antibody conjugation: A standard buffer was prepared by mixing 0.5% (v/v) of Tween-20 and 1% (w/v) of BSA in 0.01 M PBS. This standard buffer was used as a solvent for all further studies. A stock solution of conjugated CdSe QDs was prepared by mixing QDs with antihuman IgG antibody (Ab) to a final concentration of 100 nM for QDs and 200 μg mL^−1^ for Ab on a continuous shaker for 30 min.

### 2.2. Raman Spectroscopy and Fluorescence Measurements

All Raman and fluorescence intensity measurements were performed at room temperature using a Renishaw inVia micro spectrometer (Gloucestershire, UK) equipped with a 532 nm excitation laser with a laser power of 1 mW at 10× magnification. All measurements were performed in triplicate and averaged to obtain the final spectrum.

### 2.3. Fluorescence Microscopy

Fluorescence images at different stages of the experiment were captured using a Zeiss Axio Imager A1 upright microscope (Carl Zeiss Microscopy GmbH, Jena, Germany) equipped with a 120-watt metal halide lamp as a light source and an Axiocam MRc 5 camera for live image recording. All imaging was carried out under Zeiss Filter set 20 (488020-0000) with excitation of 546 nm and emission in the range of 575–640 nm with 20×/40× objectives.

### 2.4. Time-Resolved Fluorescence Measurements

A Fluorolog^®^-3 spectrometer (Horiba Scientific, Kyoto, Japan) with a NanoLED 375L pulsed laser source was used for all TRPL studies. The detector monochromator was set at 655 nm with a 15 nm bandpass for data acquisition. A custom 3D-printed sample holder was used to mount the sample substrate inside the instrument.

### 2.5. Electrical Measurements

Following the desiccation of Streptavidin/Ab-conjugated QD on the graphene surface, the electrical measurements on the SLG-FET device were carried out using the portable printed circuit board (PCB) readout system. Two Keithley 2400 (Tektronix, Beaverton, OR, USA) source/measure units (SMU) were used to measure the source-drain current *I_ds_* as a function of the gate voltage *V_g_*. For the measurements, the source-drain voltage (*V_ds_*) was kept biased at 100 mV while the gate was swept in the −1.5–1.5 V window while measuring the *I_ds_*. A 500 ms delay was applied between the source and measurement to stabilize the applied gate voltage (step size-1 mV). For all measurements, a dormancy period of 5 min. was maintained following the analyte dropcast to ensure the proper ion redistribution within the electrolyte droplet.

## 3. Results

**Figure 1:**
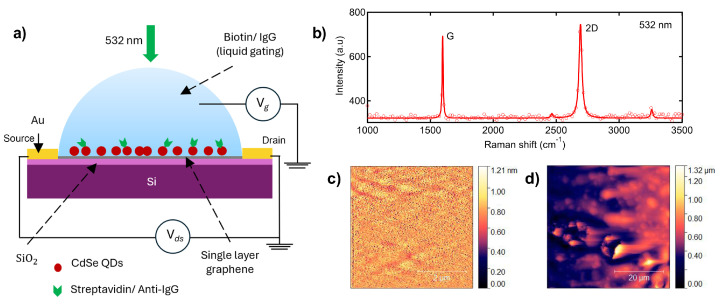
(**a**) A schematic representation of the SLG-FET device and the measurement scheme showing both optical and electrical detection. While a 532 nm incident laser excites the QDs for optical detection, transfer characteristics are simultaneously measured using the FET channels. (**b**) Raman spectrum of the chemical vapor deposition grown pristine SLG sample obtained under 532 nm excitation shows a 2D band that is more intense than the graphitic or G band consistent with SLG. The solid line shows the fit to the experimental spectrum. AFM images of the graphene sample (**c**) before and (**d**) after CdSe QD deposition show the increase in average surface roughness from 90 pm to 120 nm.

Our graphene-based sensing platform uses highly photoluminescent QDs pre-coated with detection antibodies that are deposited on the surface of SLG (Figure 1). Initially, PL emission from QDs is quenched by SLG. When the analyte binds the detection antibody, quenching is disrupted, and QD PL is restored, enabling analyte detection in a single step. We hypothesize that the quenching and restoration of QD PL is closely linked to the charge transfer (prior to optical excitation) between SLG and QDs [8,11]. In order to simultaneously investigate both PL quenching and charge transfer, we adopted the experimental approach shown in Figure 1a by using SLG-FETs for both optical and electrical characterization.

The Raman spectra of single-layer graphene (SLG) on Si/SiO_2_ (Figure 1b) exhibited the characteristic sharp graphitic (G) and double-resonant harmonic of in-plane transverse optical phonon (2D) peaks. The ratio of integrated intensities of the 2D and G band (*I_2D_/I_G_*) was found to be 3.2 from the Raman fits. The full width at half maximum of the 2D band, 27 cm^−1^, along with a high *I_2D_/I_G_* value, confirms the purity of SLG structure in the samples used in this work. The AFM images (Figure 1c,d) obtained after air drying QDs dropcast on SLG provide a qualitative comparison of the surface morphology of the samples, with the average root mean square roughness increasing from 90 pm to 120 nm, after the deposition of 10 nM CdSe QDs.

**Figure 2:**
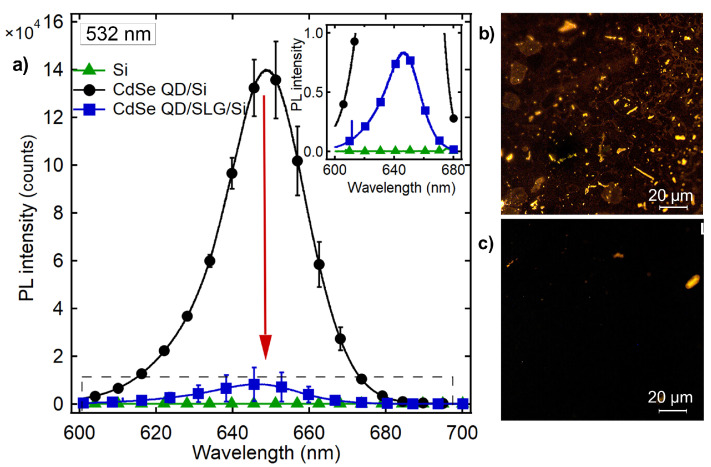
(**a**) PL emission spectra obtained from 10 nM QDs dropcasted on Si/SiO_2_ and SLG-coated Si/SiO_2_ substrates under 532 nm excitation. All data were averaged over at least three measurements. The background PL emission from bare Si/SiO_2_ is also shown (green line). A magnified view of the quenched emission is shown in the inset. The quenching of CdSe QD PL on the SLG surface is clearly seen from the fluorescence microscopy images of QDs on Si/SiO_2_ and SLG-coated Si/SiO_2_ substrates (**b**,**c**).

CdSe QDs on an SLG exhibited a ∼94% decline in PL intensity when compared to those on a bare Si/SiO_2_ substrate. Such a decrease is often attributed to FRET or non-radiative transfer of an excited electron–hole pair from the emitter (donor) to an absorbing medium (acceptor) through near-field interactions [17,34]. Steady-state luminescence microscopy images also show a significant reduction in the visibility of CdSe QDs on Si/SiO_2_/SLG/QDs (Figure 2b,c). TRPL analysis of CdSe QDs deposited on quartz and SLG revealed a significant quenching effect in the presence of graphene. The normalized PL decay curves were fitted to a triexponential function

(1) $$F\left(t\right)=\sum_{i=1}^{3}A_{i}e^{\frac{-(t-t_{0})}{\tau_{i}}},$$

yielding three characteristic lifetimes (τ_1_, τ_2_ and τ_3_) and their corresponding pre-exponential coefficients ($A_{1}$, $A_{2}$ and $A_{3}$). A bi-exponential fit was found to be insufficient in accounting for the long PL decay observed in our experiments. Previous studies suggested that a triexponential fit is often required due to multiple factors such as the distribution in the lifetimes of QDs and lifetime blinking [35,36,37,38]. On quartz, the short-lived component τ_1_ exhibits a lifetime of ∼2.5 ns with a pre-exponential factor A_1_ of ∼0.98, while the intermediate (/long)-lived components τ_2_ (/τ_3_) decay with a lifetime of 120 ns (/2500 ns) and an amplitude A_2_(/A_3_) of 0.021 (/0.002). In contrast, when CdSe QDs are deposited on SLG, A_1_, A_2_ and A_3_ were significantly reduced to 0.86, 0.003, and 0.001 respectively, indicating strong static quenching. The quenching efficiency for each type of decay process is given by

(2) $$Q_{E}^{\left(i\right)}=1-\frac{A_{\mathrm{SLG}}^{\left(i\right)}}{A_{\mathrm{Quartz}}^{\left(i\right)}},$$

where *i* is the order of the decay process. $Q_{E}^{\left(2\right)}$ and $Q_{E}^{\left(3\right)}$ were found to be ∼0.8 and 0.45 for the second and third decay processes, suggesting significant static quenching due to the presence of SLG. However, $Q_{E}^{\left(1\right)}$ for the shorter relaxation (τ_1_) remained small (0.12), indicating a lesser contribution to the overall PL quenching.

**Figure 3:**
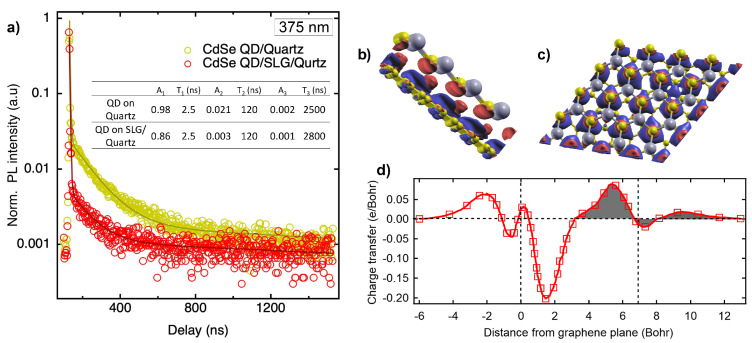
(**a**) Normalized TRPL traces of (**a**) CdSe QDs deposited on bare quartz and SLG-quartz substrates. Solid lines are obtained through triexponential fits. The fit parameters are shown in the inset. Measurements were taken by dropcasting 5 µL of 10 nM QDs onto the substrate, followed by overnight ambient drying. (**b**,**c**) Three-dimensional images of CdSe/SLG hybrids from Quantum Espresso with charge depletion (/accumulation) shown in blue (/red) color and (**d**) charge transfer profile along the *z*-axis. The total amount of charge transfer between the graphene and the CdSe layer was calculated by integrating the shaded area.

Interestingly, the τ_1_ and τ_2_ of QDs on SLG did not show significant changes, suggesting that a substantial fraction of QDs undergoes charge transfer while the remaining emissive population experiences no modifications in its radiative recombination pathways. This behavior is consistent with a static quenching mechanism, wherein QD excitation is suppressed due to charge transfer between QDs and SLG prior to optical excitation rather than an increase in non-radiative decay rates typically observed in dynamic quenching. Given that PL decay arises only from optically active QDs, we approximate that only 15% of the QD population remains emissive, while 85% of the QDs are quenched via charge transfer prior to excitation, based on the data in Figure 3a.

The quenching of CdSe QDs on SLG can be understood as follows: Let the basis states include the exciton state $|\Psi_{1}\rangle$ of QDs and the SLG conduction state $|\Psi_{2}\rangle$. The system Hamiltonian can be expressed as a sum of the Hamiltonians for the SLG ($H_{g}$), QD ($H_{QD}$), SLG–QD interaction ($H_{int}$), and light excitation ($H_{exc}$), as shown below.

(3) $$H_{g}=-t\sum_{\langle i,j\rangle ,\sigma }\left(a_{i\sigma }^{†}b_{j\sigma }+b_{j\sigma }^{†}a_{i\sigma }\right),$$

where *t* is the nearest-neighbor hopping parameter, and $a_{i\sigma }^{†}$ and $b_{j\sigma }^{†}$ are the electron creation operators on graphene’s A and B sublattices.

(4) $$H_{\mathrm{QD}}=\sum_{m}E_{m}c_{m}^{†}c_{m},$$

where $c_{m}^{†},c_{m}$ are the creation and annihilation operators for the exciton states in QD, and $E_{m}$ are the discrete energy levels of QD.

(5) $$H_{\mathrm{int}}=\sum_{m,k}\left(V_{mk}c_{m}^{†}d_{k}+V_{mk}^{*}d_{k}^{†}c_{m}\right),$$

where $V_{mk}$ represents the tunneling matrix element between the QD exciton states and graphene electronic states, and $d_{k}^{†},d_{k}$ are the creation and annihilation operators for graphene electrons.

(6) $$H_{\mathrm{exc}}=\sum_{m}g_{m}\left(c_{m}^{†}a_{k}+a_{k}^{†}c_{m}\right)$$

where $g_{m}$ is the coupling constant for optical excitation and $a_{k}$ is the photon annihilation operator.

In the absence of excitation, one can express the system Hamiltonian in $|\Psi_{1}\rangle$ and $|\Psi_{2}\rangle$ bases as

(7) $$H=\left[\begin{array}{cc} E_{\mathrm{QD}} & V_{mk}\\ V_{mk}^{*} & E_{k} \end{array}\right],$$

where $E_{\mathrm{QD}}$ is the exciton energy of QD, $E_{k}$ is the energy of the electronic state in graphene, and $V_{mk}$ represents the SLG–QD coupling, which facilitates charge transfer. This coupling is modulated upon the binding of biomolecules to QD–antibody conjugates through structural changes in the local environment, including reorientation of dipoles and steric hindrance.

Diagonalization of this Hamiltonian yields hybridized energy eigenvalues,

(8) $$E_{\pm }=\frac{E_{\mathrm{QD}}+E_{k}}{2}\pm \frac{\sqrt{{\left(E_{\mathrm{QD}}-E_{k}\right)}^{2}+4{\left|V_{mk}\right|}^{2}}}{2},$$

which define the modified energy states of the system due to charge transfer interactions. The corresponding eigenstates take the form

(9) $$|\Psi_{+}\rangle =\cos\theta |\Psi_{1}\rangle +\sin\theta |\Psi_{2}\rangle ,$$

(10) $$|\Psi_{-}\rangle =-\sin\theta |\Psi_{1}\rangle +\cos\theta |\Psi_{2}\rangle ,$$

where the mixing angle θ satisfies

(11) $$\tan\left(2\theta \right)=\frac{2|V_{mk}|}{E_{\mathrm{QD}}-E_{k}}.$$

The mixing of the QD and SLG states is controlled by θ. We note that molecular dipoles introduced by the analyte–antibody complex, which can generate local electrostatic fields, can shift the relative alignment and the mixing of energy levels between QDs and graphene. If $V_{mk}$ = 0, then θ =0, meaning the eigenstates remain purely QD or SLG states. If $V_{mk}\gg \left|E_{QD}-E_{k}\right|$, then θ approaches 45°, meaning the QD exciton state is strongly mixed with SLG conduction states. In these new energy eigenstates, $|\Psi_{+}\rangle$ and $|\Psi_{-}\rangle$, the exciton is partially or fully delocalized into graphene based on $V_{mk}$. As a result, QD PL is quenched, because the exciton is not confined in the QD long enough for radiative recombination.

The charge transfer rate is given by Fermi’s golden rule as

(12) $$\Gamma_{\mathrm{CT}}=\frac{2\pi }{\hbar }\sum_{k}{\left|V_{mk}\right|}^{2}D\left(E_{k}\right)\delta \left(E_{\mathrm{QD}}-E_{k}\right),$$

where $D(E_{k})$ is the density of states of graphene. When is $V_{mk}$ high, the charge transfer time $\tau_{CT}=\frac{1}{\Gamma_{CT}}$ is very rapid, particularly when $E_{\mathrm{QD}}$ aligns with any available electronic states in graphene.

The probability that a QD is excited before charge transfer occurs is given by

(13) $$P_{\mathrm{exc}}=\frac{\Gamma_{\mathrm{exc}}}{\Gamma_{\mathrm{exc}}+\Gamma_{\mathrm{CT}}},$$

where $\Gamma_{\mathrm{exc}}$ is the photon absorption rate. When $\Gamma_{\mathrm{CT}}\gg \Gamma_{\mathrm{exc}}$, charge transfer dominates, and most QDs do not undergo optical excitation, leading to the quenching of PL emission. However, the decay time remains unchanged (*cf*. Figure 3a) because only the fraction of QDs that remain optically active contribute to the observed lifetime, confirming that the quenching is most likely driven by charge transfer. Our DFT study of the CdSe-SLG stacked system revealed the formation of a charge transfer (Figure 3b,c). As shown in the charge transfer profile (Figure 3d) along the axis orthogonal to the CdSe-SLG plane (*z*-axis with SLG at *z* = 0 ), the total amount of charge transferred between SLG and CdSe was found to be 0.18e after integrating the shaded area.

The introduction of an analyte molecule into the QD–SLG hybrid system alters $V_{mk}$, leading to weaker hybridization and a lower $\Gamma_{\mathrm{CT}}$ or a higher $P_{\mathrm{exc}}$, resulting in the recovery of PL emission. Any charge transfer between the analyte and graphene also alters the QD–SLG hybridization and results in a shift in the Dirac point of the transfer curve. If the analyte donates electrons, graphene experiences an increase in electron density, shifting the Dirac point toward more negative gate voltages. Conversely, if the analyte acts as an electron acceptor, graphene becomes hole-doped, shifting the Dirac point toward more positive gate voltages. If the analyte molecules form a dipole layer on the graphene surface, they can introduce an effective gate potential that alters the charge carrier density without direct doping. The combination of these effects significantly changes the transfer characteristics of the SLG-FET, enabling the detection of molecular interactions through electrical transport measurements. Accordingly, we performed electrical measurements on SLG-FET in a liquid gating configuration (*cf*. Figure 1a).

**Figure 4:**
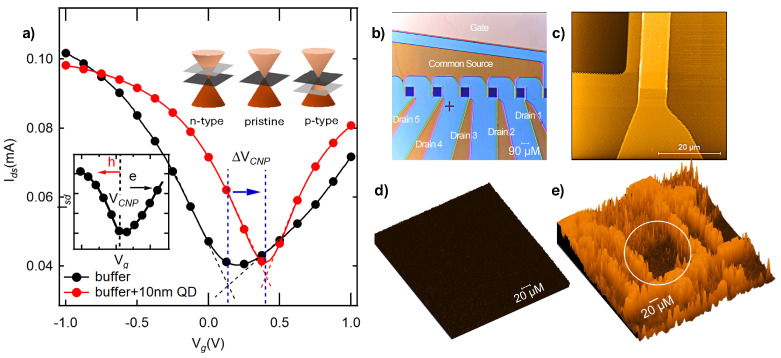
(**a**) Typical transfer curves showing $I_{ds}-V_{g}$ of our SLG-FET before and after CdSe QD deposition. The deposition was carried out by dropcasting 5 µL of 10 nM QD in the standard buffer. The inset at the bottom displays typical ambipolar transfer characteristics, showing that the type of carriers in graphene can continuously be modulated from holes (left branch) to electrons (right branch) by varying the gate voltage. The charge-neutrality point (CNP) is located at the transition between the electron and hole regime, where the current is minimum. A schematic illustration of the shift in the Fermi level across graphene’s Dirac cone upon *n-* and *p*-type doping is shown in the top inset. (**b**) Optical microscopy and (**c**) AFM images of the SLG-FET device showing graphene channels with common drain and individual sources. Contacts were made using a non-encapsulated Au gate channel, enabling liquid gating. Fluorescence microscopy images before (**d**) and after (**e**) QD deposition show strong QD luminescence everywhere except on the SLG area within the circular area (middle dark region).

The typical ambipolar transfer characteristics of the SLG-FET are shown in Figure 4a. The electron (right) and hole (left) branches extend linearly from the CNP following the relation $I_{\mathrm{ds}}=g_{m}\left(V_{g}-V_{\mathrm{CNP}}\right)$ [39,40]. The pristine SLG channel with the standard buffer exhibited $V_{CNP}$∼0.25 V, indicating that it is slightly *p*-doped. In general, $V_{CNP}$ > 0 V (/<0 V) indicates p-doping (/n-doping), with holes (/electrons) being majority carriers.

The corresponding optical, AFM, and fluorescence images of pristine SLG-FET are shown in Figure 4b–d. We did not observe any PL with pristine SLG. However, some PL was observed (from areas where QD is not on the SLG channel) after the deposition of QDs (Figure 4e). A positive shift in $V_{CNP}$ (∼0.25 V) was observed for QDs deposited on SLG, implying increased p-doping or a downshifted Fermi level (Figure 4a inset). Such a positive shift in $V_{CNP}$ concurs with our DFT calculations (cf. Figure 3b–d).

**Figure 5:**
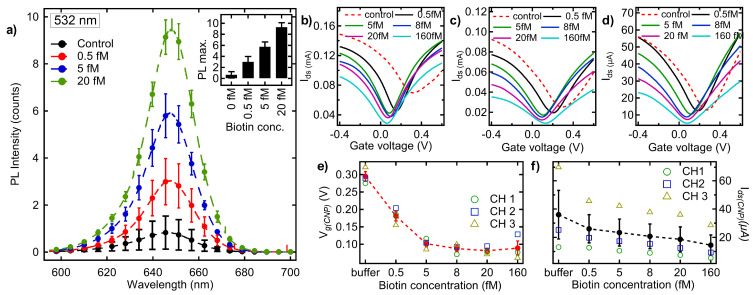
(**a**) PL Emission from streptavidin-conjugated CdSe QDs deposited on SLG increased with the addition of biotin. PL measurements were performed in triplicate for each biotin concentration. The inset shows the maximum PL intensity as a function of biotin concentration. (**b**–**d**) A lateral shift in the transfer characteristics (*V_CNP_*) was observed upon the addition of biotin to streptavidin QDs on SLG-FET. All the FET measurements were also performed in triplicate on three different SLG-FET channels shown in (**b**–**d**). Sample deposition was carried out by dropcasting 4 µl of biotin samples prepared in the standard buffer. The shift in average (**e**) $V_{C}NP$ and (**f**) *I_ds_* at *V_CNP_* as a function of biotin concentration ranging from 0.5–160 fM with buffer solution as the background control. A saturation of the sample sensitivity is observed for concentrations above 20 fM.

Leveraging the effects of charge redistribution between SLG and QDs, we performed sequential optical and electrical characterization of different analytes on the SLG-FET surface (Figure 5). We used streptavidin-coated QDs to evaluate the binding with biotin, as biotin–streptavidin is known to be one of the strongest non-covalent biological interactions. The variation in biotin concentration from 0.5 fM to 20 fM showed a linear recovery of the PL intensity (up to 70%), which can be attributed to the binding of biotin (analyte) to streptavidin (antibody)-coated CdSe QDs (Figure 5a) disrupting the charge transfer. Figure 5b–d shows the transfer curves (*I_ds_-V_g_*) recorded from three independent transistor channels (cf. Figure 4b) of streptavidin-CdSe QD-immobilized SLG-FET devices. The polarity of the CNP point (*V_CNP_*) exhibited a shift to negative voltages with increasing biotin concentration. The interaction between biotin–streptavidin QDs and SLG disrupts the overall charge distribution restoring *V_CNP_* towards 0 V due to the n-doping effect (Figure 5e). These interactions result in an upward shift in graphene’s Fermi level, which in turn enhances the net carrier density, consistent with the relation, $n\propto (V_{G}-V_{CNP})$, where *n* represents the charge carrier density.

A concomitant decline in *I_ds_* at the neutrality point was observed across all three channels (Figure 5f). For an SLG channel of length *l* and width *w*, the current modulation can be expressed as $I_{ds}=\frac{w}{l}C_{EDL}\left(V_{g}-V_{CNP}\right)V_{ds}$, where *e* is the electron charge, $C_{EDL}$ is the electrical double-layer capacitance and $V_{ds}$ is the source-drain voltage [8,12,41,42]. The observed decrease in $I_{ds}$ can be thus be attributed to either a decline in *n* or μ or a combination of both. Based on our TRPL results, which showed no change in recombination times, it is likely that the increasing biotin concentration results in the formation of localized charge puddles that decrease the electric current.

**Figure 6:**
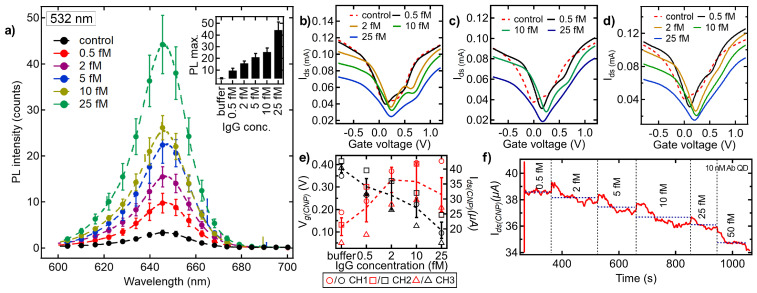
Sensing characteristics of our SLG-FET device functionalized with anti-IgG antibody-coated CdSe QDs for sensing IgG (0.5–25 fM). (**a**) PL emission at different IgG concentrations. All measurements were performed in triplicate. The inset shows the maximum PL as a function of IgG concentrations (**b**–**e**). Electrical characteristics were measured similarly to biotin. In contrast to biotin, IgG exhibits a positive shift due to *p*-type doping. All measurements were performed on at least three different SLG channels (**f**). Real-time monitoring of *I_ds_*(*V_g_* = 0.3 V) demonstrates the ability of SLG-FET to rapidly detect changes on SLG surface upon antigen–antibody binding, highlighting its potential as a robust biosensor.

To validate the feasibility of the SLG-FET system in the ultrasensitive detection of specific biomarkers, we recorded the optical and electrical response with different IgG concentrations. For this, the SLG surface was modified with anti-IgG Ab-conjugated CdSe QDs. IgG is one of the most abundant proteins in human serum, consisting of two γ heavy (H) chains and two κ or λ light (L) chains, interconnected by inter-chain disulfide bonds. The specific Y-shape with identical arms provides the antigen-binding site for antigen–antibody interactions [43]. IgG plays a vital role in the human immune system by defending against most bacterial and viral infections. Any fluctuations in IgG levels can be used as a direct indication of immune deficiencies, chronic infections, or inflammatory diseases, making it a critical biomarker in immunological diagnostics. Furthermore, simple sensors to measure IgG can also help determine the effectiveness and duration of protection following vaccination. As shown in Figure 6a, we observed a clear recovery of PL with increasing IgG concentrations. The corresponding electrical measurements on SLG-FET were able to detect IgG concentrations as low as 0.5 fM (Figure 6b–e). Unlike the biotin–streptavidin complex, we observed a further positive shift in *V_CNP_* on all three channels, indicating increased p-type doping. Our SLG-FET sensor is able to sense femtomolar changes in IgG concentration in real time in terms of gradual step decline in *I_ds_* as a function of time (Figure 6f).

**Figure 7:**
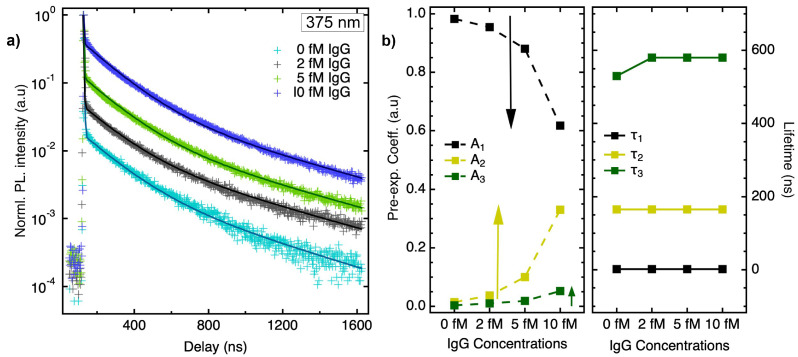
(**a**) TRPL traces for different IgG concentrations (0–10 fM) added to anti-IgG-conjugated CdSe/SLG/Quartz surface. The data were fitted to triexponential, which are shown as solid lines. (**b**) A detailed comparison of all pre-exponential amplitudes and decay times obtained from the fits.

TRPL measurements (Figure 7a) with different concentrations of IgG on anti-IgG-functionalized CdSe QDs/SLG further validated the lack of changes in decay times with increasing concentrations of IgG. As shown in Figure 7b, the amplitudes of the second and third exponential decay processes corresponding to $A_{2}$ and $A_{3}$, which are primarily dominant in SLG quenching (*cf*. Equation (2)), were found to increase concomitantly with increasing IgG concentration, while the decay times mostly remained unchanged. Based on this observation, it may be asserted that the binding of IgG to anti-IgG-coated QDs disrupts the charge redistribution at the QD–SLG interface, resulting in an increase in the emissive QD population rather than alterations in the distance between QDs and SLG.

## 4. Conclusions

This study provides a comprehensive investigation of PL quenching and recovery mechanisms in graphene–QD hybrid systems, with implications for ultrasensitive biosensing applications. Using a combination of SLG-FET measurements, TRPL spectroscopy, and DFT calculations, we demonstrate that charge transfer, rather than Förster-like resonance energy transfer, is the dominant quenching mechanism. The unaltered PL lifetime upon quenching, combined with theoretical charge redistribution analysis, supports a static quenching model governed by rapid electron transfer to graphene prior to optical excitation. Building on these findings, we successfully applied the graphene–QD platform for molecular detection, demonstrating its ability to sense biotin–streptavidin interactions and IgG biomarkers at femtomolar concentrations. Using the standard IUPAC definition as the limit of detection or LOD (3σ/slope), we estimate the LOD to be 0.9 fM for IgG detection and 0.1 fM for biotin based on slope of PL intensity as a function of concentration. The systematic shifts in the Dirac point of the SLG-FET, correlated with PL recovery, provide a robust dual-mode detection strategy that integrates optical and electrical readouts. These results establish a fundamental understanding of charge transfer interactions in graphene-based hybrid nanostructures and open pathways for developing next-generation biosensors with exceptional sensitivity, selectivity, and real-time monitoring capabilities.

## Data Availability

The raw data supporting the conclusions of this article and Supplementary Materials will be made available by the authors on request.

## Supplementary Materials

The following supporting information can be downloaded at: https://www.mdpi.com/article/10.3390/bios15050269/s1, Figure S1: Absorption profile of CdSe QDs and SLG on a quartz substrate. obtained from UV-visible spectroscopy; Figure S2: A plot of carrier concentration (*n*) as a function of $V_{G}-V_{\mathrm{CNP}}$, obtained using $\left(n=\frac{C_{EDL}\left(V_{G}-V_{CNP}\right)}{e}\right)$ assuming an electrical double-layer capacitance $C_{\mathrm{EDL}}∼2\;\mu F\;{\mathrm{cm}}^{-2}$; Figure S3: A schematic of the SLG-FET used in this study. Contacts 9 and 18 are used for liquid gating while the droplet was placed over the region shown in a dashed rectangle; Figure S4: I_*ds*_ as a function of time before and after QD deposition. To minimize background effects, a standard 5-min dormancy period was used after each sample drop-cast to ensure equilibration. The baseline over extended durations showed negligible drift within the time frame of each experiment ( 20 min); Table S1: TRPL fit parameters: calculated average lifetimes (simple amplitude-weighted), relative quantum yields (QY) for QDs on quartz, and QDs on SLG/Quartz. Section S2.1: Estimated statistical parameters from various performed experiments. References [44,45] are cited in the Supplementary Materials.

## Abbreviations

The following abbreviations are used in this manuscript:

QDs	Quantum dots
SLG	Single-layer graphene
PL	Photoluminescence
TRPL	Time-resolved photoluminescence
FET	Field-effect transistor
RET	Resonance energy transfer
DFT	Density functional theory
IgG	Immunoglobulin G
