# Supplementary material for: Unraveling Charge Transfer Mechanisms in Graphene–Quantum Dot Hybrids for High-Sensitivity Biosensing
Source: Biosensors (Basel). 2025 Apr 24;15(5):269. doi: 10.3390/bios15050269 (PMC12109218; doi:10.3390/bios15050269)
Supplement: Supplementary file 1 [file biosensors-15-00269-s001.zip › biosensors-3578094-supplementary.pdf]

Unraveling Charge Transfer Mechanisms in Graphene–Quantum Dot Hybrids for High-Sensitivity Biosensing

Shinto Mundackal Francis, Hugo Sanabria, and Ramakrishna Podila*
Department of Physics and Astronomy, Clemson University, Clemson, SC, USA

S1 Methodology

S1.1 Atomic force Microscopy:

Surface morphology of SLG-FET device was analyzed using AFM workshop TT-2 with 50 x 50 x 17 μm scanner and Budget sensor Tap190Al-G cantilevers of tip radius <10 nm and λ_{res}= 190 kHz in vibrating mode. All scans were carried out in dry conditions and were analyzed via Gwyddion image analysis software.

S1.2 Density Functional theory (DFT):

An estimation of the charge transfer between graphene and CdSe QDs was performed using DFT-D2 approach. The calculations were performed using the Quantum ESPRESSO package with ultrasoft pseudopotentials for a plane wave basis set. The generalized gradient approximation (GGA) was applied with the Perdew-Burke-Ernzerhof (PBE) exchange-correlation functional, incorporating London dispersion interactions. Exact parameters, including cell dimensions, k-mesh, and energy cutoff for the plane wave basis, were obtained beforehand by optimizing each system individually. System staking was achieved by placing 4×4 supercell size (16 Cd and 16 Se atoms each) of CdSe system over 7×7 supercell size (98 carbon atoms) of graphene.

S2 Results

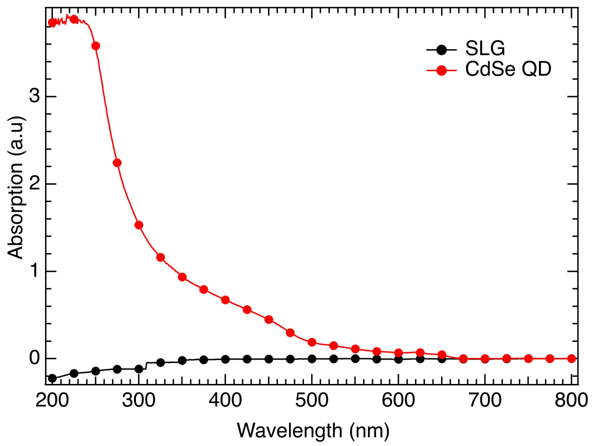

Figure S1. Absorption profile of CdSe QDs and SLG on a quartz substrate. obtained from UV-visible spectroscopy.

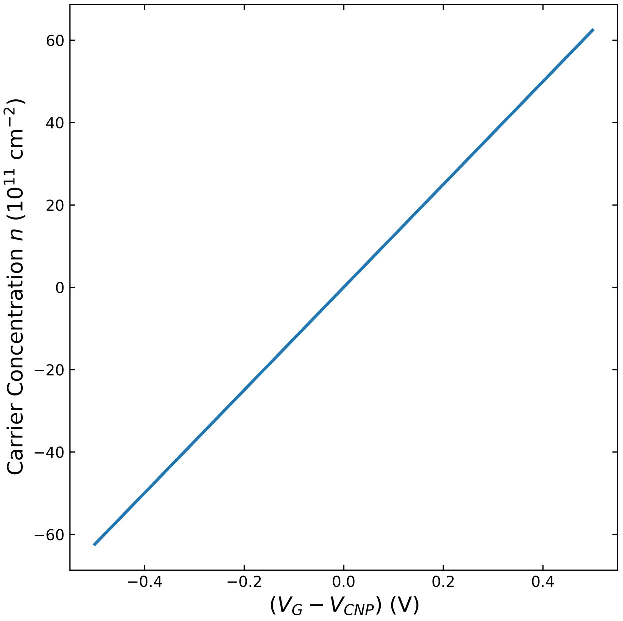

Figure S2. A plot of carrier concentration (n) as a function of $V_G - V_{\mathrm{CNP}}$, obtained using $n = \frac{C_{EDL}(V_G - V_{CNP})}{e}$ assuming an electrical double-layer capacitance $C_{\mathrm{EDL}} \sim 2\,\mu\mathrm{F\,cm}^{-2}$[1].

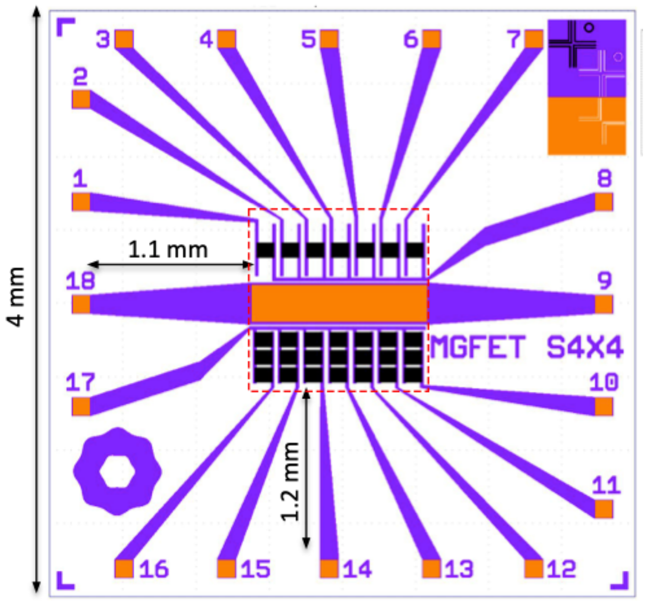

Figure S3. A schematic of the SLG-FET used in this study [2]. Contacts 9 and 18 are used for liquid gating while the droplet was placed over the region shown in a dashed rectangle.

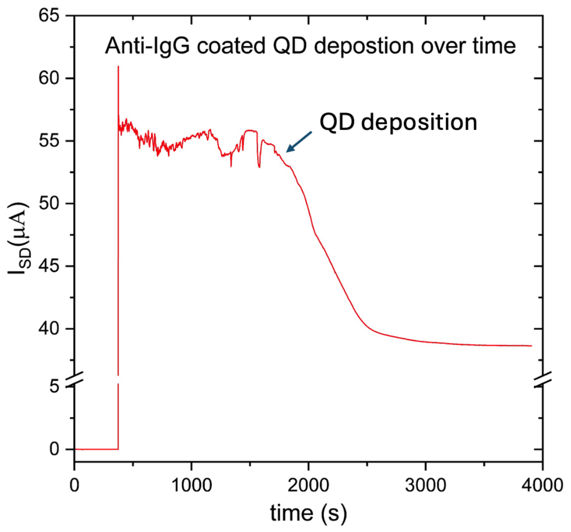

Figure S4. I_{ds} as a function of time before and after QD deposition. To minimize background effects, a standard 5-minute dormancy period was used after each sample drop-cast to ensure equilibration. The baseline over extended durations showed negligible drift within the time frame of each experiment (20 min).

Table S1: TRPL fit parameters: calculated average lifetimes (simple amplitude-weighted), relative quantum yields (QY) for QDs on quartz, and QDs on SLG/Quartz.

Sample	A_1	T_1 (ns)	A_2	T_2 (ns)	A_3	T_3 (ns)	Avg τ (ns)	Relative QY (%)
QD on Quartz	0.977	2.5	0.021	120	0.002	2500	9.9625	
QD on SLG/Quartz	0.86215	2.5	0.0035	120	0.0011	2800	6.527	65.5

S2.1 Statistical quantities

Table S2: PL intensity for different biotin concentrations with average and standard deviation corre-sponding to the error bars shown in Figs. 5 and 6

Biotin Concentration (fM)	Avg	SD
0	6572.5	5796.6
0.5	29926.4	9908.1
5	57603.0	8701.6
20	93014.6	8172.2

Table S3: V_{CNP} (V) values for different biotin concentrations measured across three channels (CH1, CH2, CH3) with average and standard deviation

Biotin Concentration (fM)	CH1 (V)	CH2(V)	CH3(V)	Avg(V)	SD (V)
Buffer	0.275	0.289	0.321	0.295	0.014
0.5	0.185	0.204	0.155	0.181	0.014
5	0.116	0.104	0.085	0.102	0.009
8	0.071	0.093	0.099	0.088	0.008
20	0.078	0.094	0.071	0.081	0.007

Table S4: I_{ds} (mA) values for different biotin concentrations measured across three channels (CH1, CH2, CH3) with average and standard deviation (rounded to 3 decimals)

Biotin Concentration (fM)	CH1(mA)	CH2(mA)	CH3(mA)	Avg (mA)	SD (mA)
0.5	0.013	0.025	0.070	0.036	0.017
5	0.013	0.020	0.046	0.026	0.010
8	0.011	0.017	0.042	0.023	0.010
20	0.009	0.015	0.038	0.021	0.009

Table S5: PL intensity for different IgG concentrations with average and standard deviation

IgG Concentration (fM)	Avg	SD
0	3067.1	543.4
0.5	9768.1	2025.3
2	15821.2	2102.3
5	21193.1	3178.4
10	25539.2	3526.8
25	44433.1	6862.6

Table S6: V_{CNP} (V) values for different IgG concentrations measured across three channels (CH1, CH2, CH3) with average and standard deviation

IgG Concentration (fM)	CH1(V)	CH2(V)	CH3(V)	Avg(V)	SD (V)
Buffer	0.187	0.132	0.051	0.123	0.039
0.5	0.237	0.301	0.087	0.208	0.063
2	0.371	–	0.222	0.332	0.056
10	0.408	0.404	0.241	0.324	0.083
25	0.416	0.177	0.208	0.267	0.075

Table S7: I_{ds} (mA) values for different IgG concentrations measured across three channels (CH1, CH2, CH3) with average and standard deviation

IgG Concentration (fM)	CH1 (mA)	CH2 (mA)	CH3 (mA)	Avg (mA)	SD (mA)
Buffer	0.043	0.038	0.040	0.040	0.001
0.5	0.039	0.031	0.031	0.034	0.003
2	0.036	–	0.026	0.031	0.005
10	0.032	0.028	0.021	0.027	0.003
25	0.025	0.019	0.015	0.019	0.003

S3 References

1. C. Hébert, E. Masvidal-Codina, A. Suarez-Perez, A. B. Calia, G. Piret, R. Garcia-Cortadella, X. Illa, E. Del Corro Garcia, J. M. De la Cruz Sanchez, D. V. Casals, E. Prats-Alfonso, J. Bousquet, P. Godignon, B. Yvert, R. Villa, M. V. Sanchez-Vives, A. Guimerà-Brunet, J. A. Garrido, *Adv. Funct. Mater.* **2018**, *28*, 1703976. https://doi.org/10.1002/adfm.201703976.

2. https://www.graphenea.com/collections/buy-gfet-models-for-sensing-applications/products/mgfet-4d-for-sensing-applications
